# 

**Published:** 1882-01

**Authors:** 


### BOOKS AND PUBLICATIONS.

"Practical Hints on the Selection and Use of the Microscope," is the title of a neat little octavo book of 238 pages. The author is John Phin, Ph. D., one of the very best authorities on microscopical science. The book is intended more for beginners, and really contains all that is worth knowing about the microscope and its accessories. We have read the book from beginning to ending, gathering much instruction therefrom, and unhesitatingly and cheerfully recommend it as one of the very best of our books upon the microscope. The modest price asked for it, places it within easy reach of all. Price $1. Industrial Publication Co., Publishers, New York.

Hand-Book of Systematic Urinary Analysis, chemical and microscopical, for the use of physicians, medical students, and clinical assistants, by Frank M. Deems, M. D., Ph. D., is the title of another very useful little book, published by the same company, as above, at $1. Everything necessary in the way of instruction in making urinary analysis is contained within the covers of this complete little volume. We commend it to physicians.

THE MICROSCOPE.—This bi-monthly journal of microscopy has reached its fifth number, and has improved steadily and decidedly with every issue. The current number contains a well executed portrait of Chas. A. Spencer, and is, an excellent likeness of that famous optician. The article on mounting microscopic objects, by Mr. Walmsley, is alone worth more than the price of the journal, for a year. Dr. Stowell is making an exceedingly interesting and useful journal of the *Microscope*. Subscription price $1 per year. Address C. H. Stowell, M. D., Ann Arbor, Mich.

POWELL'S BEEF, COD LIVER OIL AND PEPSIN. We have had occasion to use this preparation in strumous ailments particularly in those of the eye—under our care in the Surgical Institute, and have found it much better than the usual emulsions of cod liver oil that we had been using for such cases.

